# Untargeted Metabolomics Approach Correlated Enniatin B Mycotoxin Presence in Cereals with Kashin–Beck Disease Endemic Regions of China

**DOI:** 10.3390/toxins15090533

**Published:** 2023-08-30

**Authors:** Danlei Sun, Camille Chasseur, Françoise Mathieu, Jessica Lechanteur, Pierre Van Antwerpen, Joanne Rasschaert, Véronique Fontaine, Cédric Delporte

**Affiliations:** 1 Unit of Microbiology, Bioorganic and Macromolecular Chemistry, Faculty of Pharmacy, Université libre de Bruxelles (ULB), 1050 Brussels, Belgiumveronique.fontaine@ulb.be (V.F.); 2 Unit of Pharmacognosy, Bioanalysis and Drug Discovery Unit & Analytical Platform of the Faculty of Pharmacy (APFP), Faculty of Pharmacy, Université libre de Bruxelles (ULB), 1050 Brussels, Belgium; pierre.van.antwerpen@ulb.be; 3 Kashin–Beck Disease Fund, 6953 Forrieres, Belgium; 4 Laboratory of Bone and Metabolic Biochemistry, Faculty of Medicine, Université libre de Bruxelles (ULB), 1070 Brussels, Belgium; jessica.lechanteur@ulb.be (J.L.); joanne.rasschaert@ulb.be (J.R.)

**Keywords:** Kashin–Beck disease, mycotoxins, metabolism, Enniatin

## Abstract

Kashin–Beck disease (KBD) is a multifactorial endemic disease that only occurs in specific Asian areas. Mycotoxin contamination, especially from the *Fusarium* spp., has been considered as one of the environmental risk factors that could provoke chondrocyte and cartilage damage. This study aimed to investigate whether new mycotoxins could be identified in KBD-endemic regions as a potential KBD risk factor. This was investigated on 292 barley samples collected in Tibet during 2009–2016 and 19 wheat samples collected in Inner Mongolia in 2006, as control, from KBD-endemic and non-endemic areas. The LC-HRMS(/MS) data, obtained by a general mycotoxin extraction technic, were interpreted by both untargeted metabolomics and molecular networks, allowing us to identify a discriminating compound, enniatin B, a mycotoxin produced by some *Fusarium* spp. The presence of *Fusarium* spp. DNA was detected in KBD-endemic area barley samples. Further studies are required to investigate the role of this mycotoxin in KBD development in vivo.

## 1. Introduction

Kashin–Beck disease (KBD), also known as the ‘Big Bone Disease’, is a chronic, disabling disease characterized by multiple deformed bones and joints [1]. This disease is endemic in Asia, from the southeast of Siberia to Tibet, affecting the northern and central provinces of China, the north of North Korea, and some areas in Russia [2]. Its onset occurs in childhood, usually between 5 and 13 years of age [3]. 

KBD is characterized by pathological cartilage degeneration, including extracellular matrix degradation, deep zone chondrocyte necrosis, middle zone with adjacent necroptosis and apoptosis, and conjointly remodeling of cartilage with inadequate growth of articular plates. The clinical symptoms are mainly observed at the ankles, knees, interphalangeal joints, wrists, and elbows with foreshortened phalanges and deformed limbs and joints, all often seen in combination with short stature [3,4,5,6]. Patient health and quality of life are clearly affected.

However, the etiology of KBD remains unknown. More than 50 environmental risk factors have been proposed, the main ones being fungal and mycotoxin contamination and selenium deficiency [7,8,9]. In the last decade, fungal contamination was thus considered one of the multifactorial environmental risk factors. The T-2 toxin, produced by *Fusarium* spp., such as *F. poae*, *F. tricinctum*, *F. acuminatum*, and *F. sporotrichoides*, is harmful to the digestive, nervous, immune, and reproductive systems and also induces skin toxicity, hematotoxicity and carcinogenesis [10,11,12]. This mycotoxin was detected in cereals from endemic KBD areas and was shown to be able to provoke chondrocyte and cartilage damage like those observed in KBD patients [13]. However, a direct correlation between the T-2 toxin and the KBD could not be established [13,14] and *Fusarium* was seldom observed in Tibetan endemic KBD areas [7,8], suggesting that further evidence is required to claim the *Fusarium* T-2 toxin as a main etiological factor of KBD. 

**Figure 1:**
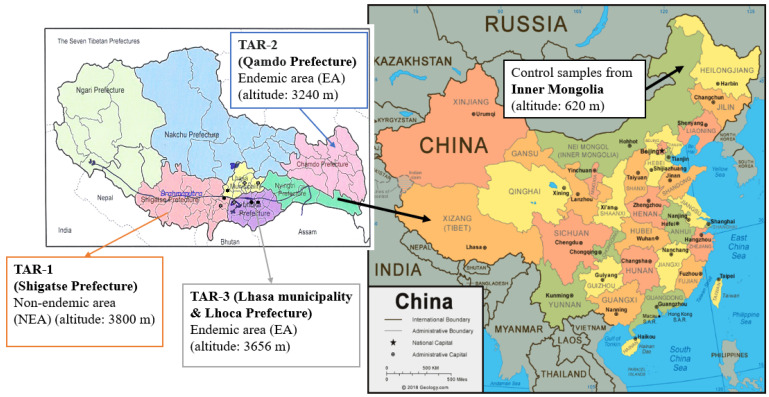
Location map for the study areas, including Tibet and Inner Mongolia in China. Barley samples collected from three different regions in Tibet include both EA and NEA between 2009–2016. Wheat samples were collected from Inner Mongolia in 2006 as controls. The China map was created by Geography.com (https://geology.com/world/china-satellite-image.shtml (accessed on 1 May 2023)).

Therefore, further epidemiological and metabolomics studies were required to investigate the involvement of other mycotoxins in the etiology of this multifactorial disease [15]. This work is the culmination of several scientific missions carried out in China for thirty years. Initially, an extension of a physical therapy program of a Belgian team of “Médecins Sans Frontières (MSF)” was carried out in the TAR (Tibetan Autonomous Region) between 1992 and 1998, allowing for the first prevalence survey of the disease in the Prefecture of Lhasa. This survey was carried out in Nyemo, Tolung, Taktse, Lundrup, and Medrogongkar counties during the period from April to July 1996 [4]. Based on a rigorous clinical diagnosis and protocol, the survey showed a prevalence rate of 11.6% for all five examined counties, ranging from 2.5% to 41.8% depending on the explored valley. Some villages even had a prevalence rate of more than 80% for the age range from 5 to 15 years old, such as the village of Parka in Medrogonkar County [4]. This region is part of the “TAR-3” KBD-endemic area (EA) involved in our study (Figure 1). Mycological analyses of stored barley from these villages allowed the detection of three common fungal taxa, *Alternaria*, *Drechslera*, and *Trichothecium*, which were significantly associated with KBD [7]. 

In 2002, MSF withdrew from Tibet, focusing on emergency humanitarian aid. The Tibetan team and international experts established the Kashin–Beck Disease Foundation (Kashin–Beck Disease Fund asbl/vzw, Belgium). From 2004 to 2008, the second clinical study (http://www.kbdfund.org/projets.html (accessed on 1 July 2023)) was conducted on an expanded territory comprising three prefectures, Lhasa, Lhoka (Shannan) and Shigatse prefectures (Figure 1). This study included various actions to prevent cereal contamination during the entire agricultural cycle, monitored by mycological analyses and the KBD prevalence study [4]. KBD prevalence was assessed by the TAR Center for Disease Prevention and Control and the Kashin–Beck Disease Foundation based on clinical evidence, in agreement with various scientific reports [16]. Beside the TAR-3 region, including Lhasa and Lhoka prefectures, well-established as KBD EA [17], the prefecture of Qamdo (TAR-2) was also investigated due to the high KBD prevalence observed in 2010 [18,19]. In 2014, the prevalence of KBD in the Qamdo district was monitored by the Centers for Disease Control and Prevention of TAR, together with the Qamdo Center for Disease Prevention and Control [15,16]. In Basu County and Luolong County, the most serious KBD-endemic areas in the Qamdo district, the KBD prevalence rate in adults reached 21.89% and 28.68%, respectively. The investigation also took place, as control, in the Shigatse prefecture (in Rimpung county), identified as a KBD non-endemic area (NEA). This area is referred to as TAR-1 in our study because of its proximity to the studied EA (Figure 1). 

In all the studied villages, barley was the main cereal and most often the only consumed cereal. In the studied TAR areas, a significant difference in grain contamination was observed with a higher prevalence of *Alternaria* spp. in EA compared to NEA; sometimes, differences were observed between families with or without KBD [7,8]. Specific and short analyses were also conducted outside the TAR to obtain more control cereals. In the North of China, surveys conducted by Chinese researchers correlated the presence of *Fusarium* spp. (*F. oxysporum*, *F. verticillioides*) with the KBD [14]. Therefore, wheat sampled in 2006 in Inner Mongolia was also analyzed as controls.

Based on the above reports, we hypothesized that unknown mycotoxins from the Tibetan endemic region may be additional risk factors that cause KBD. We took advantage of the EA and NEA cereal collections and of more recent analytical and data treatment tools [20] to identify compounds specifically or more abundantly present in the KBD-related regions, especially in Tibet. Metabolomics analysis of the data obtained by liquid chromatography coupled to a high-resolution tandem mass spectrometer (LC-HRMS(/MS)) was performed to analyze and compare the compound profiles in endemic versus non-endemic KBD areas. 

## 2. Results

### 2.1. Metabolomics Analysis

In order to analyze mycotoxins in KBD EA and NEA cereal samples, we first performed untargeted metabolomics analysis by LC-HRMS data on Workflow4Metabolomics (W4M) platform [21]. A first screening by metabolomic data analysis was performed on 27 (three replicates of nine pooled samples) Tibetan pooled samples, and on six (three replicates of two pooled samples) Inner Mongolia pooled samples (Table 1) for easier interpretation of metabolomic data. A total of 1230 features (i.e., one *m*/*z* [mass/charge] value at one retention time) were detected from the 27 pooled samples. Unsupervised principal component analysis (PCA) and univariate statistical analysis performed by MetaboAnalyst were carried out to analyze whether samples could be distinguished depending on EA and NEA, eventually on the harvested year and whether the origin of products could be determined according to the feature repartition. 

**Figure 2:**
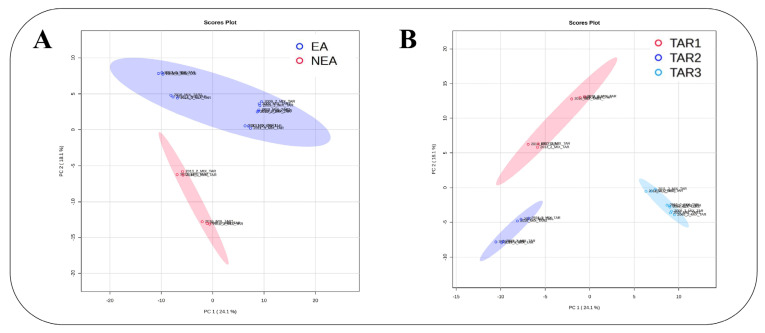
LC-HRMS metabolomics based on data post-processed with the W4M platform. Multivariate PCA modeling (performed on the MetaboAnalyst 5.0 platform) of the matrix variations in three regions (TAR-1 belongs to NEA, and TAR-2 and TAR-3 belong to EA). The score plot of the first predictive (PC 1 = 24.1%) and the second predictive (PC 2 = 18.1%) component is represented in (**A**), where EA is in blue and NEA is in red, while (**B**), where TAR-1 (NEA) is in red, TAR-2 (EA) in dark blue and TAR-3 (EA) in light blue.

Results from the PCA clearly distinguished KBD EA from NEA Tibetan samples (Figure 2A). The two first components of the PCA model accounted for 24.1% of the explained variance. Indeed, the features were scattered from both EA and NEA sample regions. Component 2 clearly separated the EA and NEA sample regions (Figure 2A), but other obvious differences were also observed, probably due to different year and geographical sampling harvests. Since EA samples were collected from two studied regions (Qamdo as TAR-2 and Lhasa municipality and Lhoca as TAR-3) geographically far away from each other, with KBD prevalence being higher in TAR-2 than in TAR-3, the PCA was performed on different sample combinations, collected either in TAR-1, in TAR-2 or in TAR-3. The PCA showed clear discrimination among the three regions, even if TAR-2 and TAR-3 were both KBD-endemic regions, with 24.1% total variance on the dataset due to the first two components (Figure 2B). 

A comparative study was similarly and simultaneously performed with wheat samples, one of the main cereals from Inner Mongolia. The results obtained with those wheat samples are clearly different (Figure S1), as expected, considering the difference in the different cereal species, the harvested year and the collected region. Meanwhile, PCA showed a clear difference between EA and NEA wheat sample data in Inner Mongolia (Figure S1). 

The Biosigner tool was used to select the variables that most are significant for the classification performances between Tibetan EA and NEA sample groups. Biosigner performs recursive elimination of features that do not significantly account for the prediction performances of binary classifiers with either the PLS-DA, Random Forest (RF) or Support Vector Machines (SVM) approaches [22]. As Biosigner works with an internal resampling approach, we only retained features that were present in four Biosigner runs. Only the ion with *m*/*z* 657.4459 at the retention time of 1026 s was interestingly retained in EA, while an ion with *m*/*z* 658.4493 at the same retention time could be a natural isotope of 657.4459. The univariate statistical analysis was performed, and a significant difference (*p*-value < 0.05) was observed between Tibetan EA and NEA samples (Figure S2). This compound was later identified as ENN B. 

### 2.2. Molecular Network Analysis

**Figure 3:**
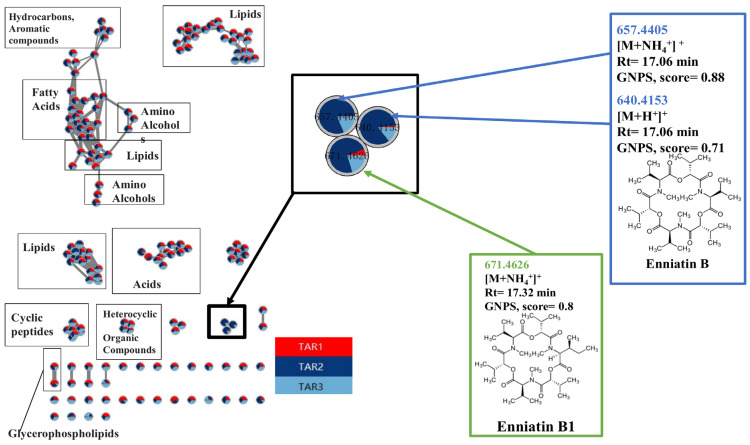
The entire molecular network of barley samples from the non-endemic area (NEA) TAR-1 and endemic areas (EA) TAR-2 and TAR-3 were analyzed by LC-HRMS/MS. Samples from each area were mixed, and three times replicates were analyzed per area and per year. Data were preprocessed by MZMine2, and the cluster annotation was performed using MetGem software. Pie charts in nodes are the proportion of the presence in each area, red, dark blue, and light blue being respectively TAR-1, TAR-2, and TAR-3. Analogs from database search implemented in MetGem software indicated Enniatin could be the candidate related to the KBD EA region in Tibet.

The molecular network analysis performed by MetGem software of the cereal pooled samples, including 160 nodes grouped in clusters (Figure 3), aimed to represent the spectral relationships among features based on their structural similarity, built by analyzing all the MS/MS data. Each node represents an MS/MS spectrum with a unique couple of *m*/*z* precursor ion and retention time values [23]. The specific similarity between two MS/MS spectra allowed us to calculate a cosine score (CS), representing the connection between nodes by the thickness of the edges. The distance between nodes is arbitrary and has no special meaning. For our study, nodes with CS higher than 0.5 and at least ten common fragment ions connected to clusters were shown on the molecular network. 

Furthermore, nodes are presented as pie charts that represent the proportion of presence in the different types of samples (Figure 3). The main influence factor was the region, and the features only present in the endemic area (TAR-2 and TAR-3, respectively, light blue and dark blue in Figure 3) were analyzed. Most of the nodes/clusters had a nearly equivalent repartition in EA and NEA, making them less interesting for our study. However, a small cluster including three nodes (highlighted in Figure 3) was only found in the endemic area due to the molecular network analysis. 

For the annotation of these nodes, the standard and analog searches were based on the interrogation of public databases (GNPS and Massbank imported in MetGem software) [24]. Several molecules were detected equally from both EA and NEA, such as fatty acids, lipids, amino alcohols, cyclic peptides and glycerophospholipids (Figure 3). The annotations included the signal at *m*/*z* 657.4405 related to other nodes having a signal at *m*/*z* 640.4153 with a CS of 0.88 and a signal at *m*/*z* 671.4626 with a CS of 0.76, while the signal at *m*/*z* 640.4153 was related to the signal at *m*/*z* 671.4626 with a CS of 0.62. This suggested a strong relationship between the features in the cluster. Based on GNPS and Massbank analog search, a group of mycotoxins with a structure similar to Enniatin B1 and B was identified (Figure 3) and further investigated (see below). The scores represented the structure similarity between annotation and molecules from the database. Furthermore, molecular network analysis performed on pooled samples from Inner Mongolia further confirmed that this molecule cluster was mainly related to the EA (Figure S3) and was also present in the EA of Inner Mongolia.

### 2.3. Identification of Enniatin B Analogs and Study of Their Distribution in EA and NEA 

According to the metabolomics and molecular networking results, two enniatin-analogs were suspected to be present only in EA. Two of the three ions, *m*/*z* 657.4433 and 640.4153, were identified as [NH4^+^]^+^ and [H^+^]^+^ adducts. The *m*/*z* 671.4570 was identified as ENN B1. 

**Figure 4:**
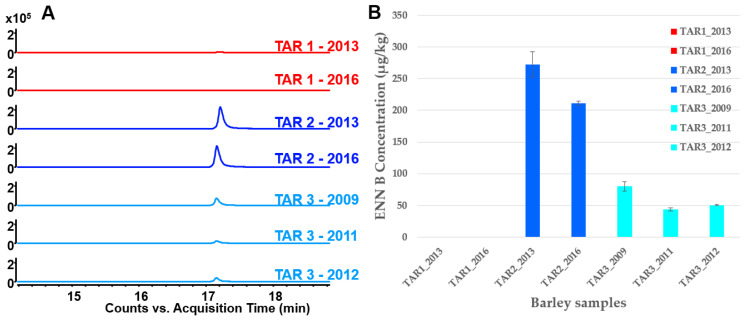
(**A**) Comparison of EIC (Extracted Ion Chromatograms of *m*/*z* 640.4139 + 657.4433 ± 50 ppm) of a few selected cereal samples from different regions and harvest years by MS analysis. (**B**) The content of ENN B detected from barley samples both in TAR-1, TAR-2 and TAR-3 (*n* = 3 for each group).

The ENN B1 with *m*/*z* 671.4570 was quite low in terms of intensity. It was only present in a few pooled and individual samples and was no longer investigated. Conversely, the two extracted ions of ENN B precursors provided from the molecular network results, observed in the chromatograms, showed ENN B was barely represented in NEA (TAR-1) but was observed in all EA (TAR-2 & TAR-3) for all studied years (Figure 4A). ENN B was highly present in the TAR-2. For a more intuitive sample comparison between different regions and harvested years, the *m*/*z* 657.4433 feature was selected for intensity comparison (Figure 4B). In TAR-1, ENN B was seldom detected at very low amounts in individual cereal samples and was not even detected in pooled samples. In TAR-2, the ENN B level was two or three times higher (average of 220 μg/kg in 28 individual samples) than in TAR-3 (average of 80 μg/kg in 20 individual samples) (Table S1). The LOD and LOQ of our method were 3 and 10 μg/kg, respectively. Intriguingly, the level of ENN B varied in both TAR-2 and TAR-3 over the harvested years. In TAR-2 samples, the level of ENN B slightly decreased between 2013 and 2016, while in TAR-3 samples, the ENN B level decreased between 2009 and 2011 and remained further constant in 2012 (Figure 4B).

SIRIUS analysis was carried out to identify the features (Figure S4) based on MS/MS data computed to match the predicted isotope pattern and fragmentation trees to assess the molecular structures with large databases [25]. The feature spectra showed molecular ion peaks of [M + NH_4_^+^]^+^ at *m*/*z* 657.4405 and [M + H^+^]^+^ at *m*/*z* 640.4139, corresponding to the ENN B molecule. The predicted structure and fragmentation similarity was 100% ENN B identical. 

**Figure 5:**
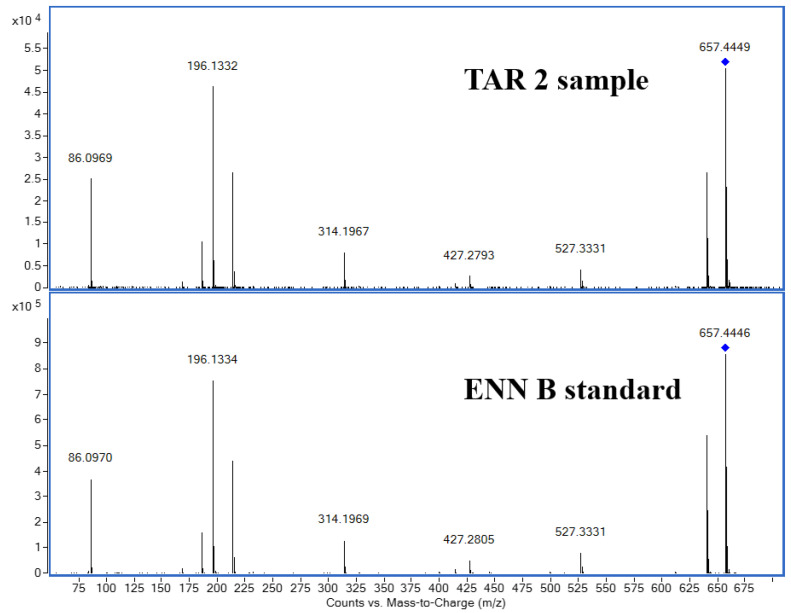
Identification of Enniatin B. Spectra comparison of barley sample from TAR-2 and standard ENN B with *m*/*z* 657.4433 as the extracted ion by MS/MS.

In order to confirm the presence of ENN B in EA samples, targeted-MS/MS analyses were further carried out to get fragmentation patterns of ions of interest corresponding to ENN B standard and on the *m*/*z* in cereal samples. The comparison of the spectra in the barley sample from TAR-2 and the ENN B standard (Figure 5) shows the same fragmentation of the extracted ion of *m*/*z* 657.44, with the same retention time between the standard and the cereal samples, in agreement with the SIRIUS fragment annotations (Figure S4). 

### 2.4. Fusarium DNA Detection in Tibetan Barley

ENN B was reported to be only produced by several *Fusarium* spp. However, these fungal species were weakly detected before by mycology analysis in Tibetan barley [7]. The presence of *Fusarium* spp. in selected Tibetan KBD EA and NEA barley samples was therefore verified by qPCR. We chose a subset of 15 barley samples among the 311 cereal samples, including 5 samples without ENN B and 10 samples with high ENN B content, according to the MS analysis (Table S2). Samples were randomly selected from the EA and NEA samples and harvested from different years to ensure that ENN B detection would be the only affecting factor. The qPCR was performed using the FusE primers, able to amplify the *Fusarium*-specific EF-1α gene elongation factor region from at least 14 *Fusarium* species, such as *F. avenaceum*, *F. oxysporum*, *F. solani*, *F. poae*, and *F. tricinctum* [26]. Some of these species are known to produce ENN B [27]. The DNA extracted from the DAOM242076 (FaLH03) *Fusarium avenaceum* strain isolated from Canadian wheat was chosen as a positive control. The linearity of detection was estimated from a standard curve with *F. avenaceum* extracted DNA, ranging from 0.14 to 14 ng/μL (Figure S5). 

**Figure 6:**
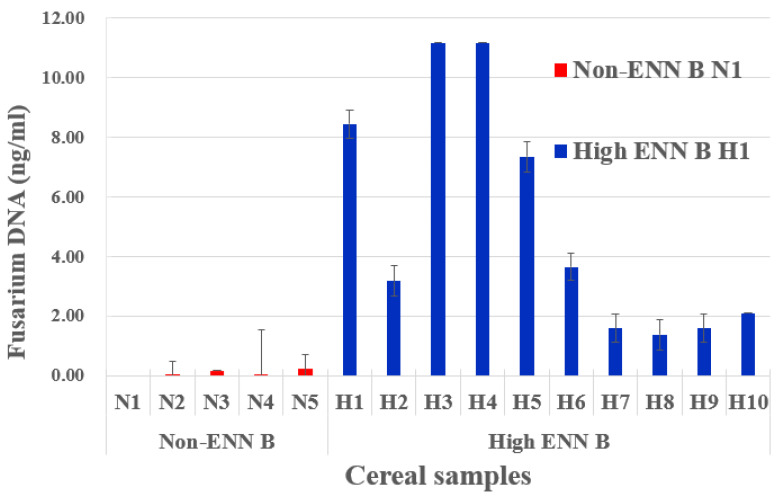
*Fusarium* DNA detection from barley samples. Content of *Fusarium EF-1α* DNA in selected barley samples with undetectable levels of ENNB (Non-ENN B, in red) and with high levels of ENNB (high ENN B, in blue), according to Tables S1 and S2.

The qPCR results showed that *Fusarium* DNA content varied substantially and accordingly between non-ENN B and high ENN B barley samples (Figure 6). Our results suggested a direct correlation between the presence of *Fusarium* DNA and the quantity of detected ENN B.

## 3. Discussion

The KBD risk factors have been analyzed by different determination methods during the past few years, but the etiology of the disease remains not understood. Indeed, the diversity of the KBD-endemic distribution in Asia, involving multiple environmental differences in terms of climate, geological, pedological, agricultural and food, further complicated the process of finding the common etiological factors. Therefore, diet was highlighted when assessing potential contamination by mycotoxins. There were approximately 1000 mycotoxins isolated from approximately 350 species of fungi. They have been chemically characterized to be classified, but only approximately 50 of them were studied in detail [28]. Moreover, mycotoxins can be produced by many fungi under specific conditions. Metabolomics aims to investigate the complete repertoire of low molecular weight metabolites, also known as the metabolome, within a biological system. Untargeted metabolomics, which aims to reproducibly measure as many as possible metabolites, is frequently used as an efficient and rapid investigation method to find out the relationship between human disease, environmental abiotic stress and microorganisms [29]. 

In this study, different metabolomic methods were chosen to highlight metabolite differences between EA and NEA for KBD cereal samples. The cereal samples were collected from different regions and years by the KBD Foundation during various scientific missions, using similar methods to allow for the most complete view of the different compounds present in EA and NEA diets. We investigated barley samples from two KBD EA (TAR-2 and 3) and one NEA (TAR-1), as barley is the main cereal in the Tibetan diet, while wheat (generally contaminated by *Fusarium*) was investigated as control from EA and NEA of Inner Mongolia. PCA analyses distinguished barley and wheat samples from EA and NEA, suggesting that some compounds, eventually arising from fungi, could be specifically or more abundantly present in EA cereals. These results were difficult to interpret because the TAR-2 samples were harvested from 2013 to 2016, while the TAR-3 samples were harvested from 2009 to 2012. After comparing the difference between the TAR-1 and the TAR-2 samples, which were both harvested from 2013 to 2016, the TAR-1 samples (NEA) presented a spread range of results because of the reported wealthier population in Shigatse prefecture compared to the TAR-2, Qamdo, population (Figure 2A,B). These observations suggested that economic development and diet improvement might have indirectly decreased KBD prevalence over the last 20 years [2]. The cereals sampling over several years was performed in order to reduce the impact of unusual environmental conditions that occur in the given year.

Multivariate analyses of the data acquired by LC-MS analyses of barley extracts were performed and could separate the EA from NEA. Thanks to these analyses and the Biosigner approach, we could identify one feature with an *m*/*z* of 657.4459 at a retention time of 1026 s as a potential feature of interest. The molecular network grouped the untargeted features by structural similarity and presented their distribution in pie charts, providing a clear and efficient way to highlight features that could be, in our case, specific to EA cereals. Only one cluster, including three nodes, was specifically present in the KBD EA barleys. Searching in MetGem and SIRIUS database and comparing MS/MS profiles with a standard ENN B, confirmed Enniatin B was mainly detected in EA. As the decrease of ENN B in TAR-3 observed between 2009 and 2011/2012 in our sample analysis took place during the Kashin–Beck Disease Foundation intervention, it is possible that our results reflect the impact of the intervention on cereal storage on the decrease of cereal fungi contamination. On the other hand, TAR-2 (Qamdo) had the highest content in 2013 and 2016, potentially reflecting the lack of sanitary measures and the absence of stored grain moisture monitoring in this area. 

Enniatin family compounds (Figure S6) are known as “emerging mycotoxins”. They are a group of structurally similar cyclodepsipeptides, produced by some *Fusarium* spp. [27]. They are generally found in a high concentration of cereals, like maize, barley and wheat, commonly cultivated in China KBD-endemic areas [30]. The ENN B average level (220 μkg) detected in TAR-2 samples is high, according to a previous study [31]. In a recent study, ENN, especially ENN B, but also ENN A, ENN A1 and ENN B1 were detected in freshly harvested Tibet barley (qingke). The contamination of ENN B in positive samples was even twofold higher than in our study [32]. This is in agreement with our study, suggesting that ENN B could be a major mycotoxin in Tibet barley. 

Although *Fusarium* were previously seldomly detected in Tibet cereals and *Alternaria*, *Cladosporium*, *Dreschlera* and *Trichothecium roseum* were significantly more detected in the KBD EA samples [7,8], we hypothesized that *Fusarium* isolation at that time was not optimal, among others because it was not performed on fresh plants [33], but on −20 °C osmobags stored cereals [7]. Indeed, our barley samples from the KBD-endemic areas were *Fusarium EF-1α* DNA positive by qPCR. Unfortunately, the ITS PCR amplicon sequencing, because of high sequence diversity, did not allow identification of the specific *Fusarium* spp. present in the barley samples. We clearly identified a correlation between the presence of ENN B in barley samples, and indirectly also barley samples with *Fusarium* contamination, and the risk of KBD in the cereal-collected areas. The results of Inner Mongolia KBD EA and NEA wheat samples showed the same correlation between ENN B and KBD-endemic area, based on the molecular networking analysis, although ENN B quantification was not performed on these samples.

Although Zhang et al. correlated the ENN B contamination level to altitude increase (alt.) as well as cumulative precipitation and temperature decrease [32], we were unable to verify this as both TAR-1 NEA samples and TAR-2 and TAR-3 EA samples were from >3000 m alt., with similar precipitation ranges (between 424–477 mm/year). Indeed, we used samples from NEA and EA, located in Tibet at >3000 m alt. or located in Inner Mongolia collected around 620 m alt. Furthermore, KBD has previously been shown not to be correlated with altitude exposition [18]. 

Several mycotoxins (T-2 toxins, butenolide and moniliformin) produced by *Fusarium* spp. were reported as potential risk factors for KBD [33,34,35]. The T-2 toxin was correlated with the wheat diet in the family that was ill with KBD [36]. The *Fusarium* spp. (*F. poae*, *F. tricinctum* and *F. acuminatum*) able to produce the T-2 toxin that are often present in wheat and maize. The *Fusarium* spp., especially *F. avenaceum*, able to produce butenolide and moniliformin, has been previously detected in barleys, even in Tibet [37,38] and can also produce ENN [27]. However, we checked for the presence of T-2 toxin in our samples, but we could not detect it. It is therefore, possible that in regions cultivating wheat and maize, KBD could arise from *Fusarium* spp. producing T-2 toxins such as *F. poae*, *F. sporotrichioides*, *F. tricinctum*, while in regions cultivating barley, KBD could arise from *Fusarium* spp. producing ENN, butenolide and moniliformin, such as *F. avenaceum*. Meanwhile, agronomic and climatic factors were reported, leading to the variation in *Fusarium* species and mycotoxins [39]. The Tibet Autonomous Region (TAR) contains specific endemic regions of KBD in China, with the highest altitude worldwide, which might be the explanation for different mycotoxin and fugal contamination compared to other KBD regions.

The toxicity of ENN compounds is based on their lipophilic nature, allowing for ionospheric properties creating cation-selective pores when they are inserted into cell membranes [40]. Enniatin B is cytotoxic for several human and animal cells when tested in vitro, eventually inducing apoptosis or necrosis, but the few in vivo studies did not allow for highlighting KBD-relevant toxicity [41,42,43,44]. This could be explained by the rapid metabolization and elimination of ENN [45]. In vivo studies demonstrated significant variations in the absorption and distribution of ENN B between broiler chickens and pigs. Broiler chickens exhibited remarkably low oral bioavailability of ENN B, with only 11% absorption [46], while pigs showed a substantially higher oral bioavailability of 91% [47]. Furthermore, broiler chickens have high total body clearance and volume of distribution for ENN [48], raising concerns about potential residues in edible tissues, such as the liver, which could potentially lead to chronic diseases upon consumption. However, chronic ENN B exposure with a broiler chicken, up to 21 days, only showed the inhibition of the enterocyte proliferation in the duodenal crypts and the limited transfer to liver tissue and plasma [48]. Nevertheless, very little data are available concerning the occurrence of ENN and its metabolites in human biological fluids [49]. Our preliminary in vitro results (not shown) suggest that ENN B is not cytotoxic up to 15 μM on human chondrocytes, a concentration higher than the concentration detected in EA barley samples. However, it is difficult to assess the risk link to ENN B for KBD. Indeed, although the ENN B concentration is low in Tibetan cereals, the amount of daily ENN B consumption could eventually reach a critical level that could influence KBD development. No studies have been performed in vivo for ENN B administration for more than 21 days. Further, in vivo studies are thus required to investigate whether ENN B, chronically administered at low concentration, eventually under selenium-deficient conditions, could be an additional risk factor, having, among others, an inflammatory activity inducing KBD-like cartilage damage [6].

## 4. Conclusions

Metabolomics approaches by both W4M and molecular network (MetGem/SIRIUS) allowed us to discriminate between samples from KBD-endemic and non-endemic areas and to identify the mycotoxins Enniatin B (ENN B) as a specific compound present in the samples from KBD-endemic regions. The presence of *Fusarium* spp. in barley samples from KBD-endemic areas was confirmed by qPCR and correlated with the presence of ENN B. Further analyses are required among other in vivo studies to eventually understand the role of Enniatin B in KBD disease.

## 5. Materials and Methods

### 5.1. Cereal Sampling

The Tibet Autonomous Region (TAR) was covered in three prefectures: Shigatse (TAR-1) as a non-endemic area, Qamdo (TAR-2), and Lhasa municipality and Lhoca (TAR-3) as the KBD-endemic areas. Shigatse is 820 km away from Qamdo, and Lhasa is just between them. In the visited Tibetan villages, barley was stored in the living areas, in bags, or in other containers. The grain sampling was performed in the second month following harvest using a double-tube grain trier with three different sampling levels. Depending on the studied regions, the harvest period differs from late August to October. In every storeroom, sample units taken in different bags are gathered to constitute one homogenous and representative sample (300 g). Cereals (wheat) were also collected in Inner Mongolia. Inner Mongolia was covered in two prefectures, including Tsa La Tsun and Hulunbuir (3245 km from Lhasa). We sampled maize, rice and wheat in 10 randomly selected families in each village. The wheat samples, which were collected directly as flour samples, were partly kept in large silos in the village and partly in bags in families. Samples were taken from the bags using the same method as for the grains in the TAR [7]. Osmobags purchased from Fisher Scientific (Hampton, VA, USA) were used to transport all grains and flour in good condition (rapid drying until 12%). The samples were stored in freezers at −20 °C [4]. The numbers of samples in each area and the years of the collection are detailed in Table 1. 

All the cereal samples from Tibet were harvested from 2009 to 2016, while the samples from Inner Mongolia were harvested in 2006, according to our activities in those prefectures, implementing projects to prevent KBD in collaboration with the Tibet CDC.

### 5.2. Metabolomics Analyses

#### 5.2.1. Materials

Acetonitrile (ACN), methanol, formic acid (FA), and ammonium formate (all LC-MS grade) were purchased from Sigma Aldrich (Steinheim, Germany). The other chemicals or reagents were of analytical grade or better. High-purified water was prepared by the Milli-Q system from Millipore (Bedford, MA, USA). Enniatin B (ENN B) was purchased from AdipoGen (San Diego, CA, USA) and stored at −20 °C in the dark. DAOM242076 (FaLH03) *F. avenaceum* (CCFC, Agriculture and Agri-Food Canada, Ottawa, ON, Canada) strain was cultured on potato dextrose agar purchased from Merck (Darmstadt, Germany) and incubated for 5 days at 25 °C before the DNA extraction. 

#### 5.2.2. Sample Preparation

A total of 311 cereal samples were collected (Table 1). After grinding into powder (15–20 s) by a mill (IKA-Werke, Staufen, Germany), 1 g of the ground cereal sample was incubated with 4 mL of extraction solution (ACN/water/FA solution; 79:20.9:0.1, *v*/*v*/*v*) for 90 min at room temperature (approximately 20 °C) in a Rotary mixer (Labinco, Breda, The Netherlands) rotating at approximately 40 rpm. After incubation, the samples were centrifuged at 2000× *g* for 10 min (Thermo Scientific, Wilmington, DE, USA). Supernatants were stored at −20 °C before analysis. Ten microliters from each cereal sample extract from the same year and region (e.g., from the 50 samples of TAR-1 collected in 2016) were mixed, giving “pooled” samples for metabolomic analysis. This process was repeated three times for each pooled sample, resulting in 27 pooled samples (3 times 9). Therefore, the initial 311 samples were grouped according to their harvest year and region, leading to 27 pooled samples. All 311 samples were also individually analyzed by the simple MS to check for the presence of ENN B. A standard solution consisting of 100 ng/mL of ENN B was prepared in the extraction solution and stored at −20 °C.

#### 5.2.3. LC-HRMS(/MS) Analysis

Analyses were performed on a 6520 series electrospray ionization (ESI)-quadrupole time-of-flight (QTOF) coupled to a 1200 series rapid resolution liquid chromatography (RRLC) from Agilent Technologies (Waldbronn, Germany). Compound separation was performed under a gradient elution on a Poroshell 120, EC-C18, 2.1 × 100 mm, 2.7 μm column with a guard column from Agilent Technologies (Palo Alto, CA, USA). The column temperature was maintained at 40 °C. The mobile phases were always composed of water with 0.1% formic acid (FA) and 5 mM ammonium formate (solvent A) and methanol with 0.1% FA and 5 mM ammonium formate (solvent B). The applied gradient was as follows: after 1 min initial time in 5% solvent B, the proportion of B was increased linearly to 90% from 1–15 min, held for 1 min, and then back to 5%, with a total runtime of 16 min; post-run 4 min. The flow was settled at 0.4 mL/min. The ESI-QTOF parameters were as follows for MS analysis: positive mode, 4 GHz resolution, MS scan range 100–3200 *m*/*z* at 1.66 spectra/s, drying gas temperature 325 °C, drying gas flow 10 L/min, nebulizer pressure 50 psi, capillary voltage 4500 V, and Fragmentor 175 V; nitrogen was used as the nebulizer gas. The reference ions, respectively, *m*/*z* 121.050873 and 922.009798, ran for QTOF continuous calibration. Analysis was carried out on MassHunter Acquisition^®^ software for QTOF (Version B.08.00) coupled with MassHunter Qualitative Analysis^®^ (Version 10.0) software (both from Agilent Technologies). Every sample was injected three times. The same quality control (QC) sample made by a mix of all samples was injected throughout the run after every ≈12 samples. Furthermore, blank (water) injections were performed ≈5 times over the run. 

For molecular network analysis and SIRIUS tools, an LC-auto MS/MS analysis was performed with the following conditions: MS scan range 100–2500 *m*/*z* at four spectra/s; MS/MS scan range 50–2500 at three spectra/s; isolation width: medium mode (~4 *m*/*z*); collision energy was 25 V; max precursors at 3/cycle; threshold 500 absolute intensities; precursor abundance-based scan speed at 25,000 counts/spectrum with MS/MS accumulation time limit; and active exclusion after three spectra released after 0.5 min.

#### 5.2.4. Data Processing and Statistical Analysis

Agilent format “.d” data were converted into “.mzXML” format using the ProteoWizard MSConvert tools (Version 3.0.20347-3e8ee6135, 64-bit) with the Peak Picking (“Vendor” algorithm) filter option with both MS and MS/MS (when both are present). 

The Galaxy workflow4metabolomics (W4M, https://workflow4metabolomics.usegalaxy.fr (accessed on 10 May 2022), Version 21.09) online platform was used for the processing of the data (peak detection, integration, filtration, peak grouping and retention time correction, and annotation), normalization, quality control (metabolites correlation analysis and batch correction), and statistical analysis (univariate testing and multivariate modeling) [21]. The entire analysis was processed on 42 samples, including mixed cereal samples (27), blank, and Quality Control (=QC) samples). All detailed parameters and data are available on the W4M platform: https://workflow4metabolomics.usegalaxy.fr/u/danlei_sun/h/unnamed-history-1 (accessed on 24 June 2023). 

Principal component analysis (PCA) and univariate analyses were also performed on the data matrix file of W4M above-mentioned analysis using MetaboAnalyst 5.0 [50] to visualize the distribution of metabolite variability and select the significance and best distinguish different groups of samples. The Biosigner tool (W4M platform) was used to find the significant features that have the best discrimination between groups. PLS-DA, random forest (RF), and support vector machines (SVM) have been run in parallel as binary classifiers by Biosigner. 

#### 5.2.5. Data Preprocessing with MZmine 2 and Molecular Network Analysis on MetGem

The “mzXML” format files of autoMSMS analyses were processed using MZmine 2.53 software [51]. The range time was filtered from 2 to 20 min for both MS level 1 and level 2. ADAP Chromatogram Builder was used to build chromatograms with a minimum group size of two scans, a group intensity threshold of 50, a minimum intensity of 10, and the *m*/*z* tolerance was fixed at 50 ppm. The chromatogram deconvolution was performed with an RT range for MS2 scan pairing of 0.5 min. Isotopic peak grouper was performed using the following parameters: *m*/*z* tolerance of 50.0 ppm, retention time tolerance of 0.2 min and a maximum charge of 2. A joint aligner was performed for the peak alignment with the following parameters: *m*/*z* tolerance of 50 ppm, weight for *m*/*z* of 75, retention time tolerance of 0.1 min and weight for a retention time of 25. A feature list row filter was performed using the same retention time range method and *m*/*z* range from 100 to 2500, the minimum peaks in a row of three and minimum peaks in an isotope pattern of 2 while keeping only peaks with an MS2 scan (GNPS). The gap-filled was performed using the peak finder with the following parameters: intensity tolerance of 10, *m*/*z* tolerance of 50 ppm, and retention time tolerance of 0.5 min. Data were then exported as “.mgf” files for spectra using Export/Submit to GNPS-FBMN and .csv files for metadata information using Export to “.csv” file. 

Molecular networks were built on MetGem 1.3.6 software. The “.mgf” file and “.csv” files were imported using the following standard parameters [23]: *m*/*z* tolerance of 0.02 and minimum matched peaks of 9. The data were filtered by the following parameters: peaks kept outside of the ±17 Th widow, peaks in the top 6 in the ±50 windows. The network visualization of the filter was the maximum neighbor number (top K) of 10, a cosine score (CS) above 0.5, and a maximum connected component size of 1000. For standard and analog searches in databases (GNPS and Massbank) [24], the parameters were as follows: *m*/*z* tolerance of 0.02, minimum matched peaks of 4, minimum intensity of 0%, *m*/*z* parent tolerance of 17 Th, and minimal cosine score value of 0.65. 

#### 5.2.6. LC–MS/MS Data Annotation with SIRIUS

To annotate features of interest, Agilent format “.mgf” of autoMSMS data were processed using SIRIUS 5.6.3 software with the following standard parameters [25]: filter by isotope pattern; MS2 mass accuracy, 10 ppm; molecular formula candidates stored, 10; formula database, ALL; and possible ionizations with [M + H^+^]^+^, [M + NH_4_^+^]^+^, [M + Na^+^]^+^, [M + K^+^]^+^.

### 5.3. Fusarium DNA Detection

#### 5.3.1. DNA Extraction

Ground cereal sample DNA were extracted with the E.Z.N.A.^®^ Fungal DNA Mini Kit (Omega BIO-TEK, Norcross, GA, USA), according to the manufacturer’s instructions. The concentrations of extracted DNA were assessed using the NanoDropTM 2000/2000C spectrophotometer (Thermo Scientific, Wilmington, DE, USA) and stored at −20 °C. 

#### 5.3.2. Quantitative PCR (qPCR) and Data Analysis

The primers FusEF (5′-CTGGGTTCTTGACAAGCTCA-3′) and FusER (5′-CGGTGACATAGTAGCGAGGA-3′) were used to amplify the *Fusarium* elongation factor *EF-1α* gene, as previously described [26]. qPCR was performed in triplicate using TakyonTM No Rox SYBR^®^ MasterMix dTTP Blue (Eurogentec, Seraing, Belgium), containing 10 μL of 2× MasterMix, 2 μL primers (100 nM), 1 μL H_2_O, and 5 μL DNA (20 ng/μL) in a final volume of 20 μL. The qPCR program, processed on the C1000^TM^ (BioRad, Temse, Belgium) with CEX96TM Real-Time PCR Detection System, was as follows: initial denaturation time of 3 min at 95 °C, followed by 39 cycles of denaturation at 95 °C for 10 s, annealing at 60 °C for 30 s, an extension at 72 °C for 30 s, and finally, a control step with a melting curve analysis (temperature range from 55 °C to 95 °C, with 0.5 °C for 5 s increment) to exclude contamination. Blank samples containing water instead of DNA were included on each 96 well-plate run to exclude the possibility of contamination.

A standard curve was made with serial dilutions of the target *Fusarium* DNA extracted from DAOM242076 (FaLH03) *F. avenaceum* (CCFC, Agriculture and Agri-Food Canada, Ottawa, ON, Canada).

## Figures and Tables

**Table 1:** Description of the cereal samples.

Cereal	Non-Endemic Area	Endemic Areas	
Barley	2013—10 samples—TAR-1	2013—37 samples—TAR-2	2009—25 samples—TAR-3
	2016—50 samples—TAR-1	2016—90 samples—TAR-2	2011—40 samples—TAR-3
			2012—40 samples—TAR-3
Wheat	2006—6 samples—IM	2006—13 samples—IM	

## Data Availability

Data are available at this link when you are logged on the W4M platform: https://workflow4metabolomics.usegalaxy.fr/u/danlei_sun/h/unnamed-history-1 (accessed on 24 June 2023), under the name “Metabolomics for cereal in Tibet” by author D. Sun.

## Supplementary Materials

The following supporting information can be downloaded at https://www.mdpi.com/article/10.3390/toxins15090533/s1, Figure S1: Principal Component Analysis (PCA) of cereal samples: red, barley from Shigatse (TAR-1); dark blue, barley from Qamdo (TAR-2); light blue, barley from Lhasa municipality and Lhoca (TAR-3); black, wheat from the EA region of Inner Mongolia; yellow, wheat from the NEA region of Inner Mongolia. Score plot of the first predictive (PC1 = 74%) and the second predictive (PC2 = 6.4%) component is represented in PCA; Figure S2: The box plot of regions and intensities for comparing EA and NEA to identify the differences of ENN B (*m*/*z* 657.4459 at 1026 s) between the two groups; Figure S3: Molecular network of specialized metabolites identified in cereal samples by LC-HRMS/MS analysis. Cluster annotation was performed using databases from MetGem. Pie charts inside nodes denote abundance, while colors correspond to the regions where the feature was found. The cluster related to TAR-1 was circled in red, the one related to TAR-2 in light blue, TAR-3 in dark blue, the NEA of Inner Mongolia in yellow, and the EA of Inner Mongolia in black; Figure S4: MS/MS data was analyzed by SIRIUS software with the pattern analysis and fragmentation comparison giving the structural similarity of molecules based on the database. The annotation of the 657.44 *m*/*z* fragment was done using the SIRIUS software. Figure S5: Standard curve obtained by qPCR of *Fusarium EF-1α* DNA (ng/μL) according to the obtained cycle threshold (Ct) values. The *Fusarium EF-1α* DNA concentration range was 0.14 to 14 ng/μL per reaction. The linear regression equation of the standard curve was Y = −2.75x + 33.833 at r^2^ = 0.992. Figure S6: Chemical structures of Enniatin [52]. Table S1: Detection of Enniatin B in barley samples. Table S2: List of barley samples with their origin (both of EA and NEA) and harvested year.
