# Supplementary material for: Untargeted Metabolomics Approach Correlated Enniatin B Mycotoxin Presence in Cereals with Kashin–Beck Disease Endemic Regions of China
Source: Toxins (Basel). 2023 Aug 30;15(9):533. doi: 10.3390/toxins15090533 (PMC10537395; doi:10.3390/toxins15090533)
Supplement: Supplementary file 1 [file toxins-15-00533-s001.zip › toxins-2496832-supplementary/Figure Supplements.pdf]

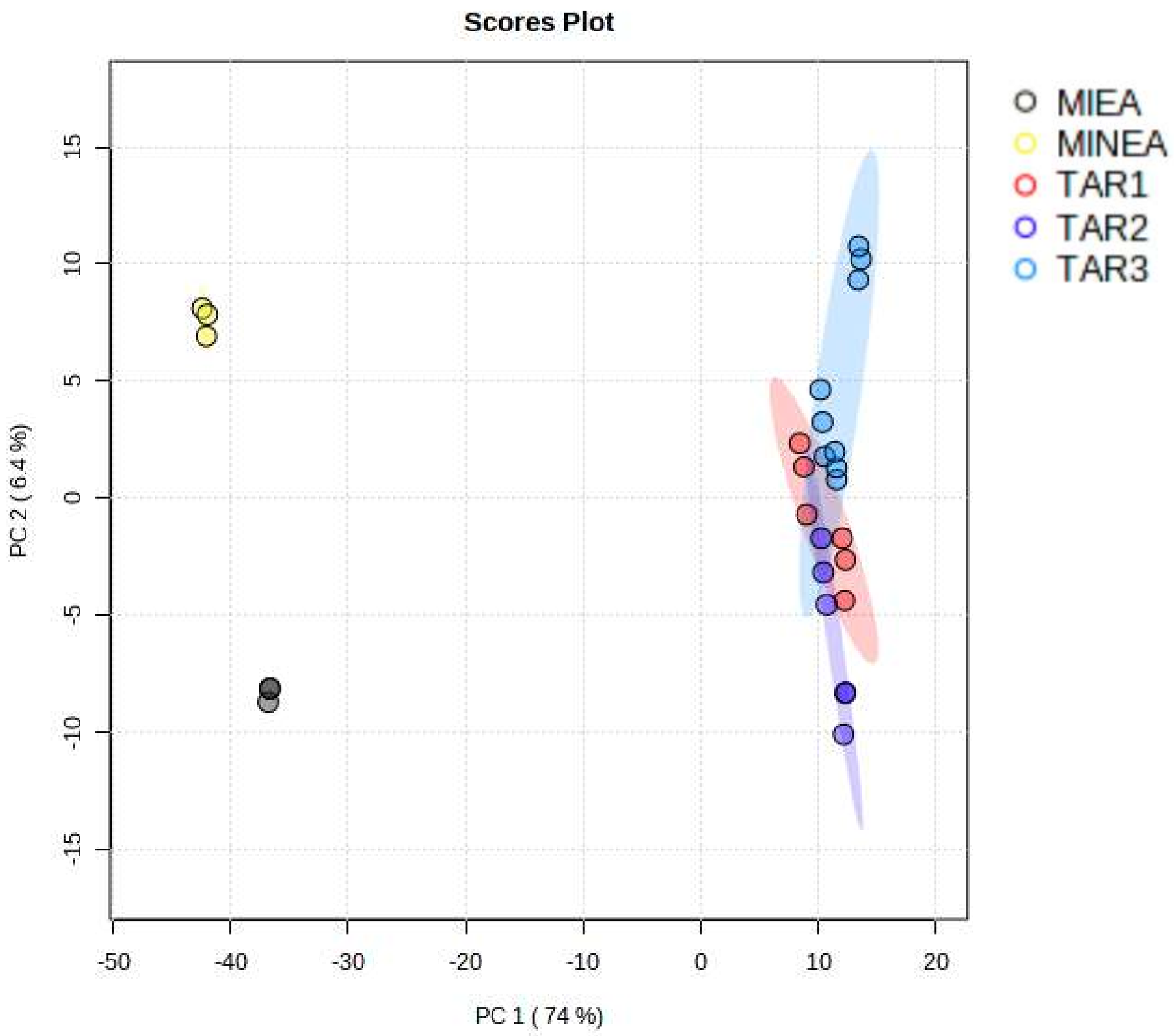
Scores Plot
PC 1 (74 %)
PC 2 (6.4 %)
-50
-40
-30
-20
-10
0
10
20
15
10
5
0
-5
-10
-15
MIEA
MINEA
TAR1
TAR2
TAR3

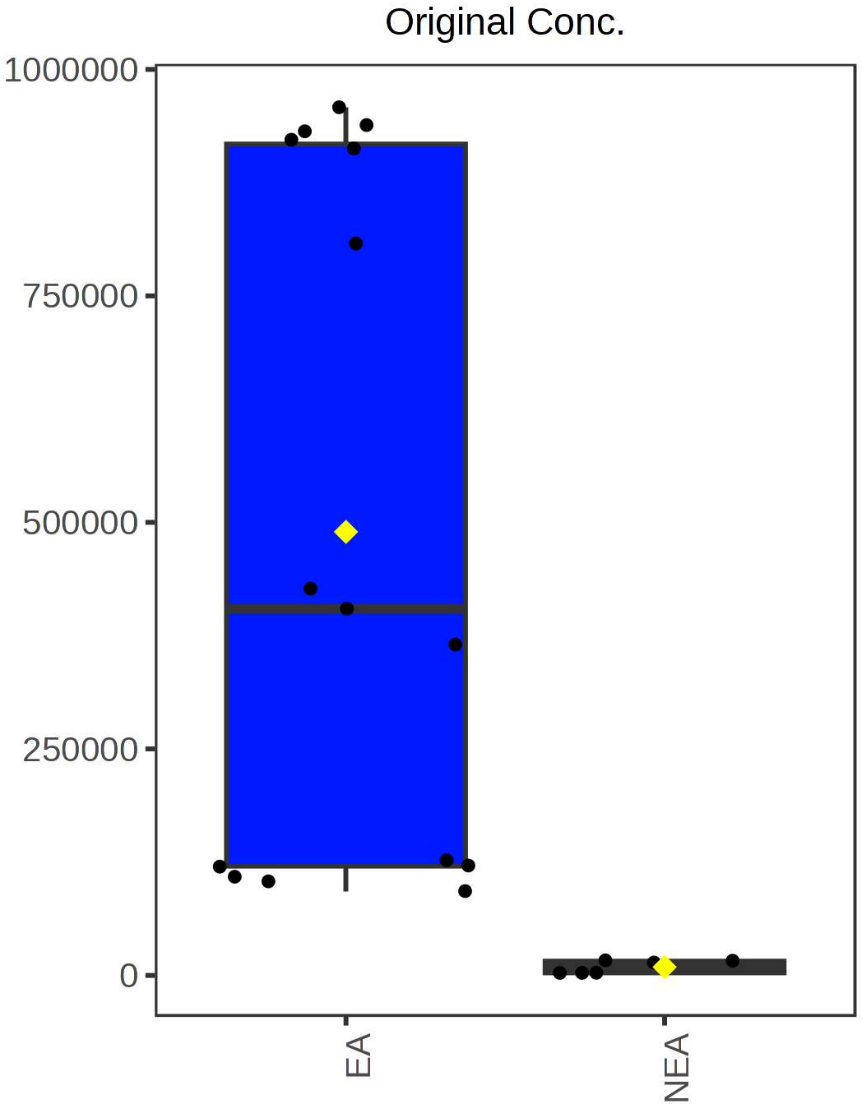
Original Conc.
1000000
750000
500000
250000
0
EA
NEA

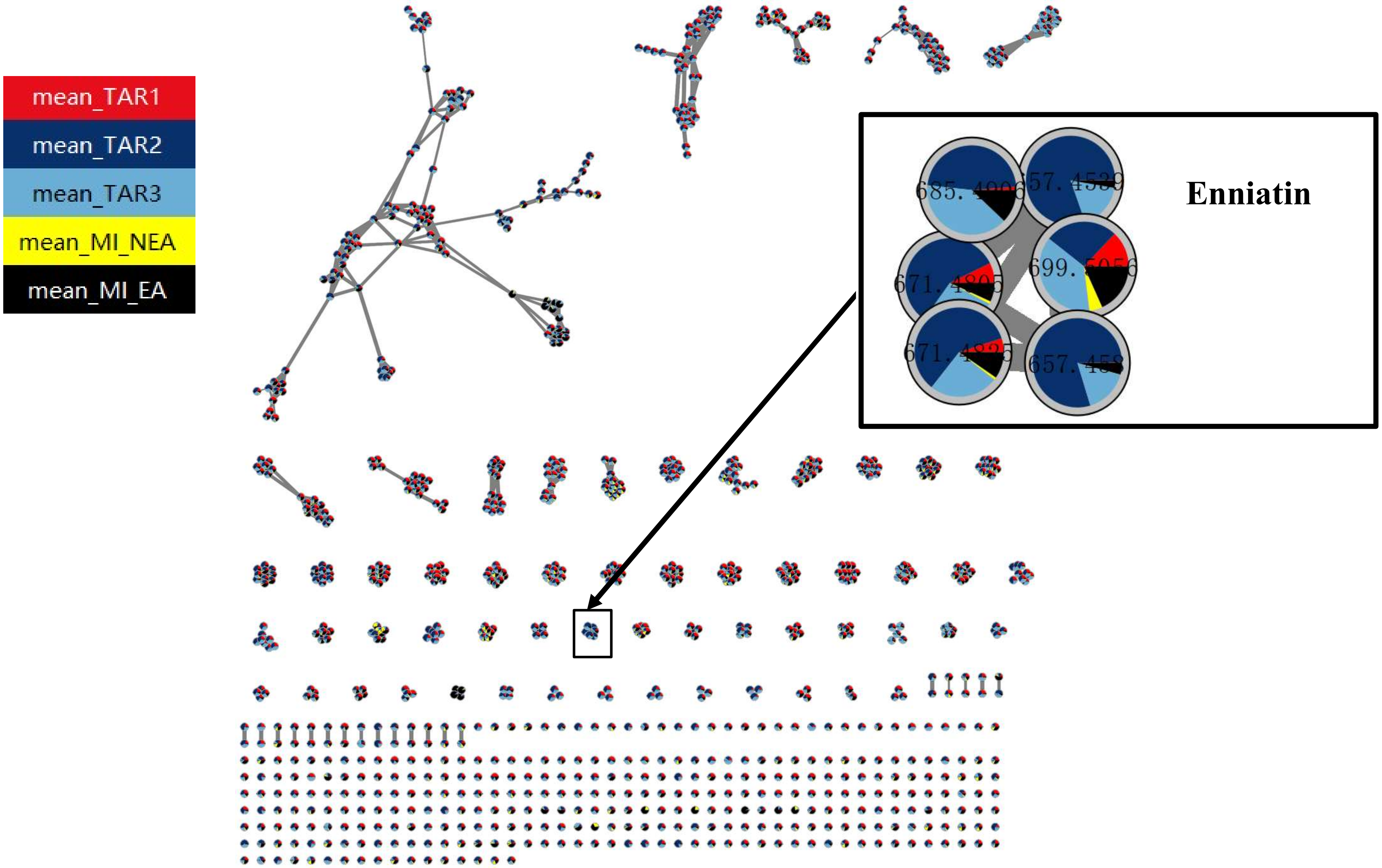
mean_TAR1
mean_TAR2
mean_TAR3
mean_MI_NEA
mean_MI_EA
Enniatin

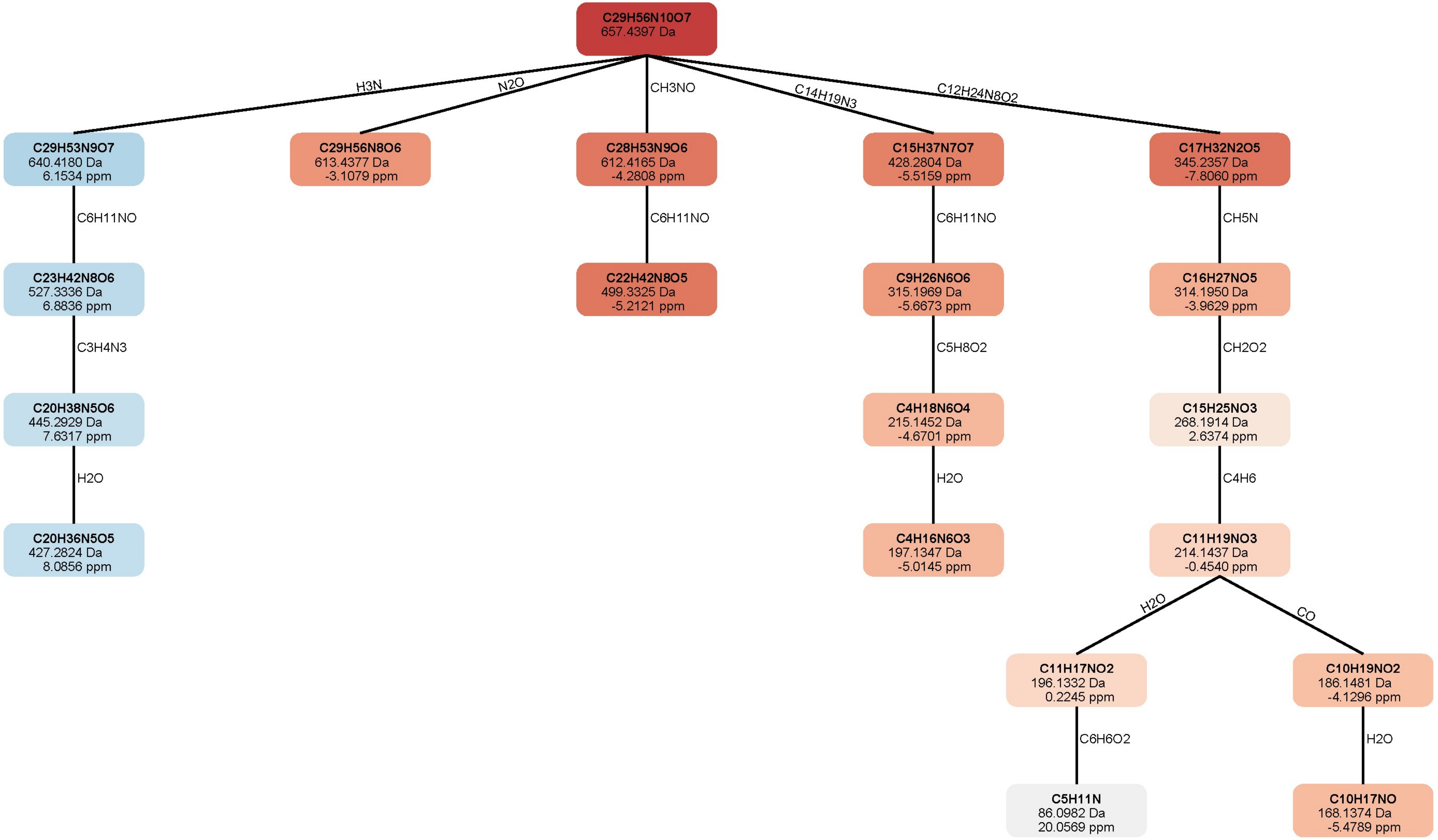
C29H56N10O7
657.4397 Da
H3N
N2O
CH3NO
C14H19N3
C12H24N8O2
C29H53N9O7
640.4180 Da
6.1534 ppm
C29H56N8O6
613.4377 Da
-3.1079 ppm
C28H53N9O6
612.4165 Da
-4.2808 ppm
C15H37N7O7
428.2804 Da
-5.5159 ppm
C17H32N2O5
345.2357 Da
-7.8060 ppm
C6H11NO
C6H11NO
C6H11NO
CH5N
C23H42N8O6
527.3336 Da
6.8836 ppm
C22H42N8O5
499.3325 Da
-5.2121 ppm
C9H26N6O6
315.1969 Da
-5.6673 ppm
C16H27NO5
314.1950 Da
-3.9629 ppm
C3H4N3
C5H8O2
CH2O2
C20H38N5O6
445.2929 Da
7.6317 ppm
C4H18N6O4
215.1452 Da
-4.6701 ppm
C15H25NO3
268.1914 Da
2.6374 ppm
H2O
H2O
C4H6
C20H36N5O5
427.2824 Da
8.0856 ppm
C4H16N6O3
197.1347 Da
-5.0145 ppm
C11H19NO3
214.1437 Da
-0.4540 ppm
H2O
CO
C11H17NO2
196.1332 Da
0.2245 ppm
C10H19NO2
186.1481 Da
-4.1296 ppm
C6H6O2
H2O
C5H11N
86.0982 Da
20.0569 ppm
C10H17NO
168.1374 Da
-5.4789 ppm

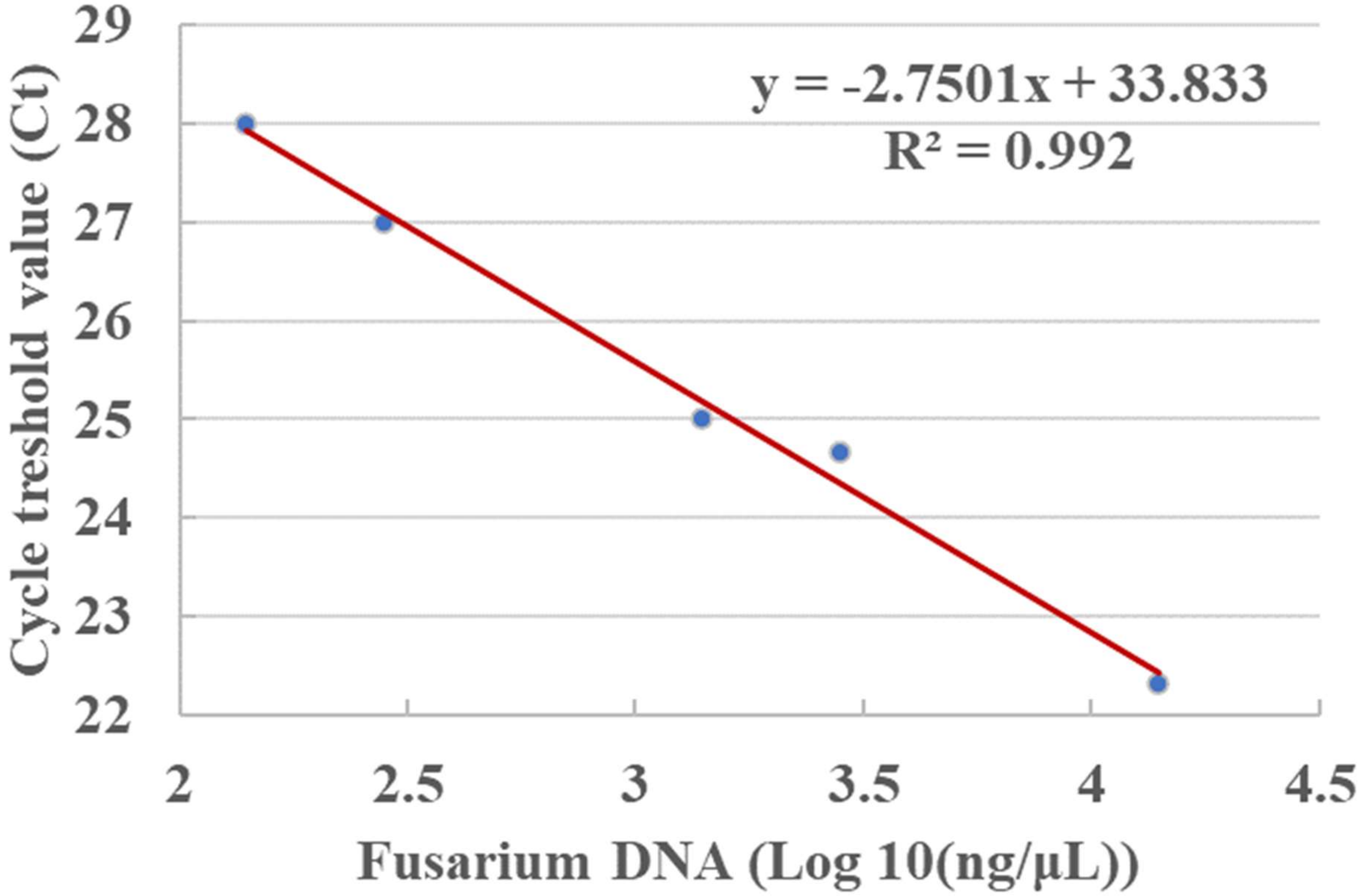
y = -2.7501x + 33.833
R² = 0.992
Cycle treshold value (Ct)
Fusarium DNA (Log 10(ng/µL))
29
28
27
26
25
24
23
22
2
2.5
3
3.5
4
4.5

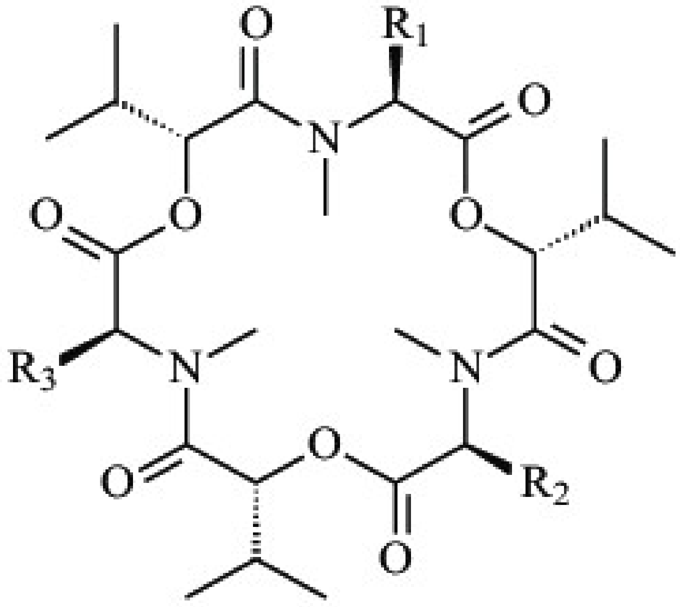

ENN	R1	R2	R3	Elemental Formula	Molecular weight (g/mol)	Exact mass (m/z)			Reference
						$[M+H]^+$	$[M+NH4]^+$	$[M+Na]^+$	
**Enniatin A**	$CH(CH_3)CH_2CH_3$	$CH(CH_3)CH_2CH_3$	$CH(CH_3)CH_2CH_3$	$C_{36}H_{63}N_3O_9$	681	682.4637	699.4903	704.4456	(Malachova et al., 2011)
**Enniatin A1**	$CH(CH_3)CH_2CH_3$	$CH(CH_3)CH_2CH_3$	$CH(CH_3)CH_2CH_3$	$C_{35}H_{61}N_3O_9$	667	668.4481	685.4746	690.4300	
**Enniatin B**	$CH(CH_3)_2$	$CH(CH_3)_2$	$CH(CH_3)_2$	$C_{33}H_{57}N_3O_9$	639	640.4168	657.4433	662.3987	
**Enniatin B1**	$CH(CH_3)_2$	$CH(CH_3)_2$	$CH(CH_3)CH_2CH_3$	$C_{34}H_{59}N_3O_9$	653	654.4324	671.4589	676.4143	
