# Supplementary material for: Untargeted Metabolomics Approach Correlated Enniatin B Mycotoxin Presence in Cereals with Kashin–Beck Disease Endemic Regions of China
Source: Toxins (Basel). 2023 Aug 30;15(9):533. doi: 10.3390/toxins15090533 (PMC10537395; doi:10.3390/toxins15090533)
Supplement: Supplementary file 1 [file toxins-15-00533-s001.zip › toxins-2496832-supplementary/Table Supplements.pdf]

Supplementary Materials: Untargeted Metabolomics Approach Correlated Enniatin B Mycotoxin Presence in Cereals with Kashin–Beck DiseaseEndemic Regions of China

Danlei Sun, Camille Chasseur, Françoise Mathieu, Jessica Lechanteur, Pierre Van Antwerpen, Joanne Rasschaert, Véronique Fontaine, Cédric Delporte

Table S1. Detection of Enniatin B in barley samples.

Sample characteristic	Sample name	Concentration (μg/kg)
**Pooled samples**	TAR1_2013	nd
	TAR1_2016	nd
	TAR2_2013	272.63
	TAR2_2016	211.02
	TAR3_2009	79.55
	TAR3_2011	43.65
	TAR3_2012	50.45
**Individual TAR1-2013 samples**	T6	nd
	T7	nd
	T8	nd
	T9	nd
	T10	nd
**TAR1-2016**	R9_2016	nd
	R17_2016	nd
	R28_2016	nd
	R31_2016	nd
	R40_2016	nd
	R44_2016	nd
**TAR2-2013**	R1	116.34
	R2	124.17
	R6	207.22
	R9	312.61
	R12	206.08
	R14	436.77
	R16	162.68
	R17	65.85
	R31	79.08
	R32	120.17
	R33	61.12
**TAR2-2016**	C3	46.56
	C5	118.06
	C6	1493.64
	C7	16.62
	C10	340.71
	C11	53.50
	C20	286.90
	C21	564.24
	C23	120.06
	C24	71.64

	C28	76.50
	C35	180.68
	C36	67.47
	C44	402.41
	C45	78.73
	C48	78.68
	C65	247.81
**TAR3-2009**	QC3_2009	283.96
	QC4_2009	73.85
	QC5_2009	19.94
	QC9_2009	80.77
	QC10_2009	344.75
	QC16_2009	18.34
	QC17_2009	74.84
	QC19_2009	24.63
	QC21_2009	70.48
**TAR3-2011**	QC20_2011	163.18
	QC29_2011	65.94
	QC32_2011	81.81
	QC34_2011	41.10
	QC37_2011	40.92
**TAR3-2012**	QC3_2012	22.45
	QC12_2012	41.91
	QC14_2012	18.30
	QC18_2012	56.08
	QC25_2012	70.94
	QC36_2012	30.00

nd (not detected), nd < LOD.

Table S2. List of barley samples with their origin (both of EA and NEA) and harvested year.

Sample group	Sample names [a]	Origin	Harvested year
Non-Enniatin B detected samples	N1; R9_2016	TAR-1-NEA	2016
	N2; R17_2016	TAR-1-NEA	2016
	N3; R40_2016	TAR-1-NEA	2016
	N4; QC16_2012	TAR-3-EA	2012
	N5; QC30_2012	TAR-3-EA	2012
High Enniatin B detected samples	H1; C6	TAR-2-EA	2016
	H2; C7	TAR-2-EA	2016
	H3; C21	TAR-2-EA	2016
	H4; C20	TAR-2-EA	2016
	H5; C36	TAR-2-EA	2016
	H6; C48	TAR-2-EA	2016
	H7; QC10_2009	TAR-3-EA	2009
	H8; R6	TAR-2-EA	2013
	H9; R31	TAR-2-EA	2013
	H10; R16	TAR-2-EA	2013

[a] Sample names mention first a short-called name for *Fusarium* DNA quantification experiment followed by the sample name used in Enniatin B quantification (Table S1).
